# Rhesus Monkeys (*Macaca mulatta*) Do Recognize Themselves in the Mirror: Implications for the Evolution of Self-Recognition

**DOI:** 10.1371/journal.pone.0012865

**Published:** 2010-09-29

**Authors:** Abigail Z. Rajala, Katharine R. Reininger, Kimberly M. Lancaster, Luis C. Populin

**Affiliations:** 1 Neuroscience Training Program, University of Wisconsin-Madison, Madison, Wisconsin, United States of America; 2 Department of Anatomy, University of Wisconsin-Madison, Madison, Wisconsin, United States of America; 3 Eye Research Institute, University of Wisconsin-Madison, Madison, Wisconsin, United States of America; 4 Department of Psychology, University of Wisconsin-Madison, Madison, Wisconsin, United States of America; Kyushu University, Japan

## Abstract

Self-recognition in front of a mirror is used as an indicator of self-awareness. Along with humans, some chimpanzees and orangutans have been shown to be self-aware using the mark test. Monkeys are conspicuously absent from this list because they fail the mark test and show persistent signs of social responses to mirrors despite prolonged exposure, which has been interpreted as evidence of a cognitive divide between hominoids and other species. In stark contrast with those reports, the rhesus monkeys in this study, who had been prepared for electrophysiological recordings with a head implant, showed consistent self-directed behaviors in front of the mirror and showed social responses that subsided quickly during the first experimental session. The self-directed behaviors, which were performed in front of the mirror and did not take place in its absence, included extensive observation of the implant and genital areas that cannot be observed directly without a mirror. We hypothesize that the head implant, a most salient mark, prompted the monkeys to overcome gaze aversion inhibition or lack of interest in order to look and examine themselves in front of the mirror. The results of this study demonstrate that rhesus monkeys do recognize themselves in the mirror and, therefore, have some form of self-awareness. Accordingly, instead of a cognitive divide, they support the notion of an evolutionary continuity of mental functions.

## Introduction

Mirror self-recognition, measured with the mark test [1], is thought to be an indicator of self-awareness [2], [3], the capacity to comprehend that one exists as an individual separate from thoughts, other individuals, and the environment. Some chimpanzees [1] and orangutans [4], like humans [5], pass the mark test and, therefore, are self-aware. Macaques, on the other hand, are thought to lack self-awareness because, with few exceptions [6], they have consistently failed the mark test and have shown persistent social responses towards mirrors [1], even after prolonged exposure [7] and training [8].

The mark test [1], the standard test for self-recognition, is performed after first exposing an animal to the mirror, during which time the behavior may change from social interactions directed towards the reflection to self-directed behaviors [1], indicating that it may have learned to recognize its reflection as its own. The actual test consists of the application of marks on the animal's face while anesthetized, then exposure to a mirror after recovery. If the animal touches the marks, acknowledging their presence on its face, it is concluded that it has passed the test and thereby verifies the observations that suggested that it recognizes itself in the mirror [1] and, therefore, is self-aware [3].

Determining that an individual of a given species, an ape or a monkey for instance, can recognize itself constitutes a monumental problem because one cannot know objectively what is the creature's cognitive process; for a human, one cannot know what he or she is thinking. The mark test is thought to provide an objective solution to this problem. By touching the mark on its face, not the mark on the mirror, the animal is thought to show not only that it has detected the presence of the mark on its face but, fundamentally, to have judged the mark as foreign to the image of itself, demonstrating, therefore, that if has a concept of self.

The results of the mark test have been used to delineate a fundamental divide in cognitive function between hominoids and all other species [9], [10], but recent evidence has called this assertion into question. Some elephants [11], dolphins [12], and magpies [13] have passed the mark test thereby demonstrating that the ability to learn to recognize one's self in a mirror has evolved independently along different branches of the evolutionary tree [13].

It is important to note that despite its objectivity and the fact that it has become a benchmark, the mark test is not free from controversy [14]. For instance, it may fail to properly measure the cognitive abilities of species that do not self-groom or rely heavily on senses other than vision [15]. Furthermore, it may share the limitations of comparative studies of cognitive function that fail to distinguish between differences in ability and differences in performance [16]–[18]. It may be possible, therefore, that the monkey has fallen on the wrong side of the cognitive divide.

Observations of two rhesus monkeys that had been prepared for behavioral/electrophysiological studies with a head implant led us to question the assertion that monkeys do not recognize themselves in the mirror and, therefore, lack self-awareness. These monkeys held mirrors and looked into them while grooming. The results of two experiments with mirrors of different sizes, reflectivity, and location confirmed our initial observations that indicate that these animals do in fact recognize themselves in the mirror.

## Results

### Initial observations

**Figure 1:**
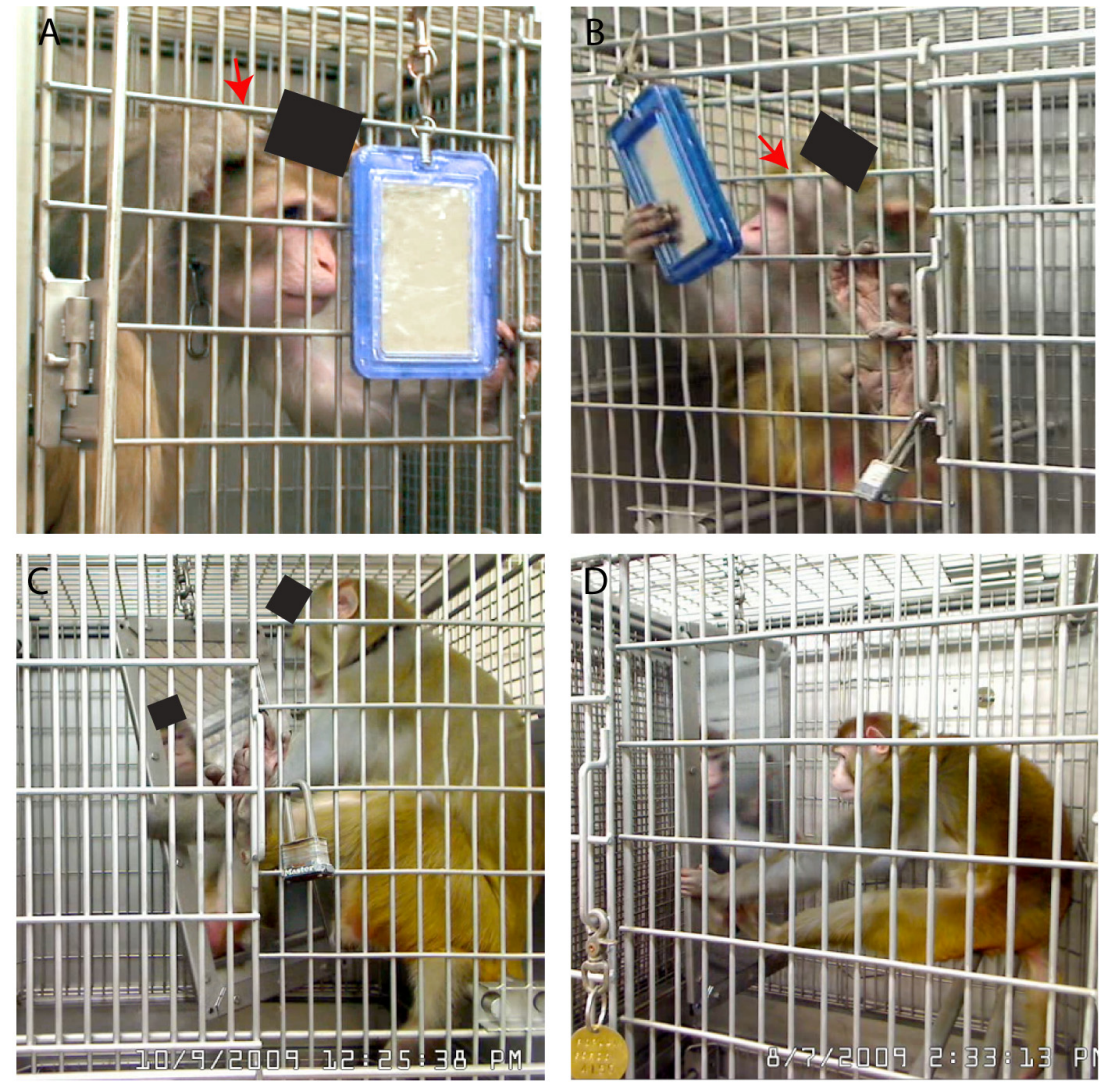
Examples of monkey self-directed behaviors in front of the mirror. (A,B) images from video recordings taken over the course of approximately eight months following initial observations. In each photograph the hand used for grooming is highlighted with a red arrow. In (A) the monkey leaned to his left while sitting on the perch to be able to look at himself in the mirror. In (B) The same monkey held the mirror at the appropriate angle for viewing himself with the right hand while grooming the area around the implant with the left. (C,D) Self-directed behaviors with the large mirror from two other monkeys. View of the implants have been masked for discretion (A–C).

Figure 1A,B shows a sample of the observations that led us to question that rhesus monkeys cannot recognize themselves in the mirror. Upon being returned to his cage after experiments this monkey moved in front of the mirror (Fig. 1A), or held it at the appropriate angle with one hand while grooming the area around the implant with the other (Fig. 1B). The images in Movie S1 (supplemental materials) illustrate that the monkey engaged in these behaviors for several minutes at a time. As reported in chimpanzees during the mark test [1], the monkey smelled, licked, and looked at his fingers while grooming in front of the mirror, indicating that he understood that the area being groomed was clearly his. Similar behaviors were observed in a second monkey. Although they occasionally groomed the area around the implant in the absence of the mirror, their gaze was not fixed in any particular location. When grooming was guided by mirror viewing, the monkeys always turned to face it and looked into it. Furthermore, there were no attempts to touch or groom the image in the mirror, which would have suggested that the monkey saw the reflection as another animal. Most importantly, no social responses were observed during the periods in which the monkeys looked at themselves and groomed in front of the mirror.

Because these behaviors had not been reported in the literature, these two monkeys were given the mark test and, consistent with previous reports [1], [7], they failed. In no instance did they show behaviors directed at the marks dyed on their faces. Thus, we had two conflicting pieces of evidence. On the one hand, both monkeys failed the mark test, which as discussed above is the standard test for self-recognition [1]. On the other hand, both monkeys exhibited behaviors that were unequivocally self-directed and guided by looking into the mirror (see Movie S1).

Since both monkeys had failed the mark test, it was imperative to design experiments around other objective measures of behavior that would allow us to determine if these monkeys exhibited self-recognition. Anderson [19] outlined the following criteria to objectively determine if an animal displays mirror self-recognition: (1) the spontaneous development of mirror-guided self-directed behaviors, such as examining parts of the body that are unseen without the aid of a mirror, and (2) the disappearance of social responses directed toward mirrors. Two experiments that included these measures were carried out to resolve the contradiction.

### Experiment 1

Although our initial observations appeared to demonstrate that monkeys use the mirror to look at and groom themselves spontaneously at any moment, the possibility existed that in response to somatosensory stimulation from being cleaned, they groomed the implant area regardless of the availability of the mirror. Accordingly, the small mirrors were removed for one week, then placed back on the cage of each of five subjects, including the two animals initially observed using mirrors to groom, for five one-hour sessions videotaped on separate days; videotaping took place before cleaning the implant area to avoid providing somatosensory cues. In addition, as a control, the animals were also videotaped in five one-hour sessions using the same mirror with the reflective surfaces covered with black plastic. We hypothesized that no differences in behavior should be observed if the reflectivity of the mirror was irrelevant and the animal was simply holding or sitting in front of an object and staring at it. Conversely, if important, the mirror should reveal self-directed actions and reduction and eventual disappearance of social responses [19].

**Figure 2:**
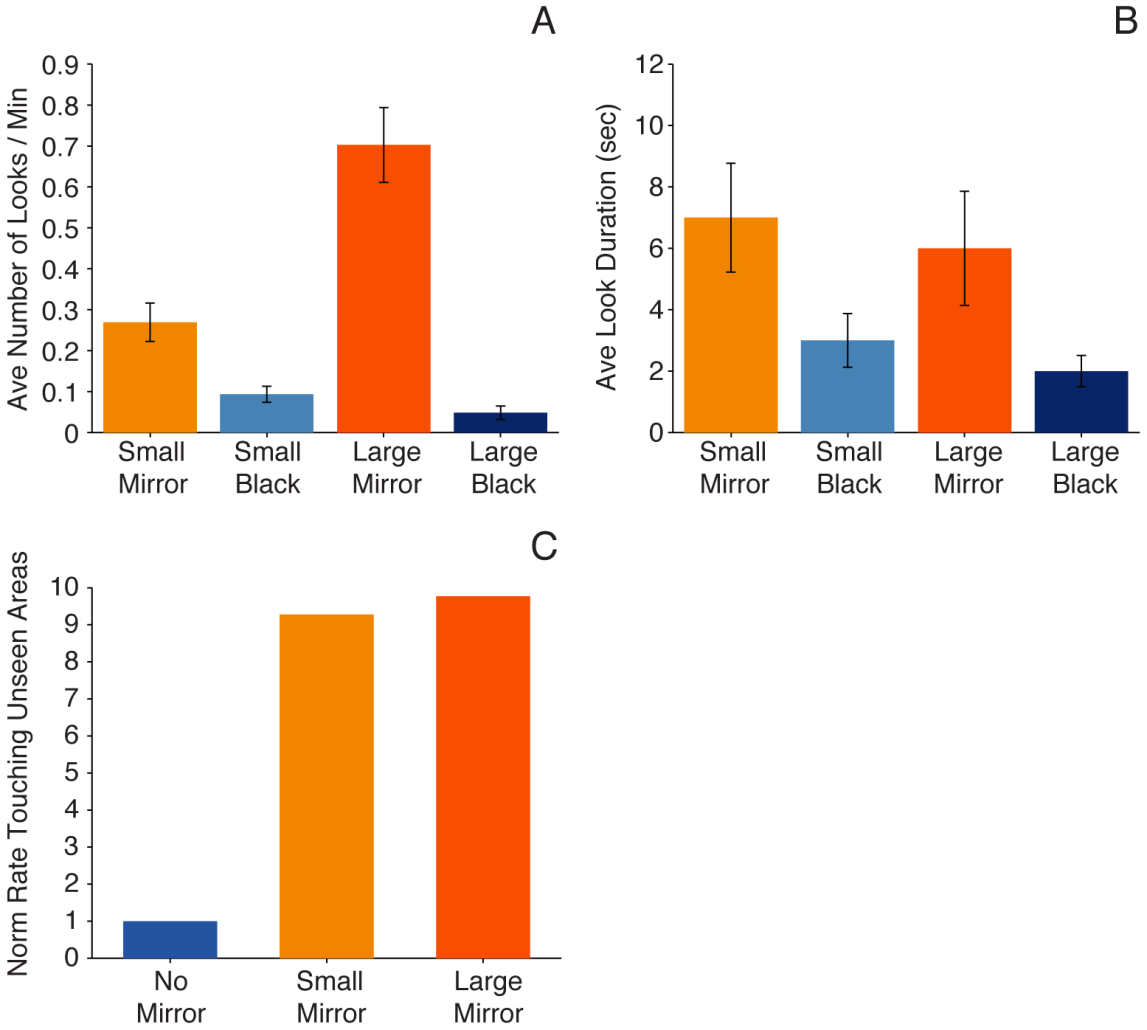
Quantification of mirror-directed behaviors. (A) Average number of looks in the mirror per minute recorded in the large and small mirror sessions and their corresponding controls covered with black, non-reflective plastic. (B) Average look duration for the mirrors and black controls. (C) Rate of touching unseen areas, the area around the implant on the head and genitals as described in B of Table 2, in the small and large mirror sessions normalized to the control. Control represents the no-mirror condition. Data from all five monkeys studied are included in this figure. All behaviors involved the monkeys moving or moving the mirror with their hands or feet to obtain the appropriate angle to look at themselves.

On average, monkeys looked at the small mirror significantly longer than the black control (*p*<0.05), approximately once every 2.5 minutes (Fig. 2A), but the duration of the looks, despite a trend for being longer than the looks into the actual mirror, was not significantly different (Fig. 2B). We hypothesize that the animals persisted in looking at the black control because it was attached to the same frame used to hold the regular mirror and stopped looking when they realized that the object was not a mirror, as revealed by the significantly larger number of looks directed at the mirror versus the control (Fig. 2A).

Except for a few instances from one of the five subjects tested, no social behaviors were observed with either the mirror or the control (Table 1). In addition, as described below, some of the monkeys used the small mirror to examine parts of the body they could not see directly. We computed the rate in which they spontaneously touched or groomed the area around the implant and other unseen areas of the body (genitals) with and without the mirror. An equal rate of touching would have indicated that the mirror was irrelevant. The data in Figure 2C indicate otherwise. The rate of touching when the mirror was present was nearly tenfold greater than without the mirror. The data have been normalized because of the small number of spontaneous touches in the control. These observations are consistent with Anderson's [19] assertions regarding behavioral events that suggest self-recognition.

**Table 1:** Average Behavior per minute or five subjects.

Behavioral Category							
	A	B	C	D	E	F	G
**Large Mirror**							
Mean	0.490	0.064	0.201	0.004	0.106	0.030	0.021
SE	0.115	0.033	0.049	0.002	0.066	0.007	0.011
**Large Black**							
Mean	0.029	0.000	0.039	0.000	0.005	0.000	0.020
SE	0.018	-	0.014	-	0.003	-	0.009
**Small Mirror**							
Mean	0.160	0.061	0.058	0.003	0.028	0.002	0.000
SE	0.042	0.018	0.017	0.001	0.016	0.002	0.000
**Small Black**							
Mean	0.046	0.007	0.043	0.000	0.003	0.000	0.015
SE	0.013	0.005	0.012	-	0.003	-	0.007

### Experiment 2

Despite the positive results of the previous experiment, the possibility existed that the small size of the mirror coupled with it being hung outside the cage may have mitigated the monkeys perceiving the images in the mirror as threatening conspecifics and thus made the observed behaviors possible. Accordingly, a large mirror in which the monkeys could see their entire body was introduced. This mirror had one reflective side, was hung in the upper half of a double space cage, and could be swiveled. It was reasoned that this arrangement would provide the monkeys room to observe themselves, inspect the backside of the mirror, or avoid it if threatened by the reflection.

The introduction of the large mirror was met with curiosity. Figure 1C,D shows two monkeys as they held the large mirror with their hands and feet while looking at themselves (see also Movie S2). The first interactions were varied and included looking behind the mirror, presumably seeking the monkey they observed in the reflection. The contingency with the mirror was quickly established, however, and social behaviors subsided during the first session.

Monkeys looked at themselves more than twice as often in the large than in the small mirror (Fig. 2A), possibly due to the novelty associated with it. This measure comprises all instances of actively looking in the mirror without social behaviors, listed under the *Self-examination* heading in Table 2. Specifically those in which the monkey turned toward the mirror, positioned it at the appropriate angle to look into it, or shifted its position to match the moving mirror in order to maintain the appropriate angle of view. In control sessions the average number of looks was smaller (*p*<0.05) than with the mirror; there were no differences in the number of looks between the controls of the two experiments (Fig. 2A). Interestingly, in control sessions two monkeys tore the cover exposing part of the mirror and looked into it intently.

**Table 2:** Behavioral categories and descriptions.

Behavior	Description
**Self-examination**	
A) Looking at himself in mirror	Specific orienting or positioning of his body in front of mirror and intent self-examination
B) Looking at himself in mirror while touching otherwise unseen areas	Specific orienting in front of mirror and grooming implant area or examining genitals
C) Looking at himself in mirror while holding it	Grabbing the mirror and specifically orienting it to self-examine
D) Looking at himself in mirror while touching otherwise unseen areas and holding it	Holding mirror in position while intently looking at reflection and grooming implant area or manipulating genitals
**Exploratory**	
E) Using mirror to look at environment (as a tool)	Angling the mirror to indirectly examine areas of the environment or neighboring conspecifics
G) Looking behind the mirror	Examining space behind or around mirror
**Social**	
F) Behaviors observed when an animal comes into contact with an unknown conspecific	Signs of aggression or submission such as charging the mirror, open-mouth threats, or lip smacking

**Figure 3:**
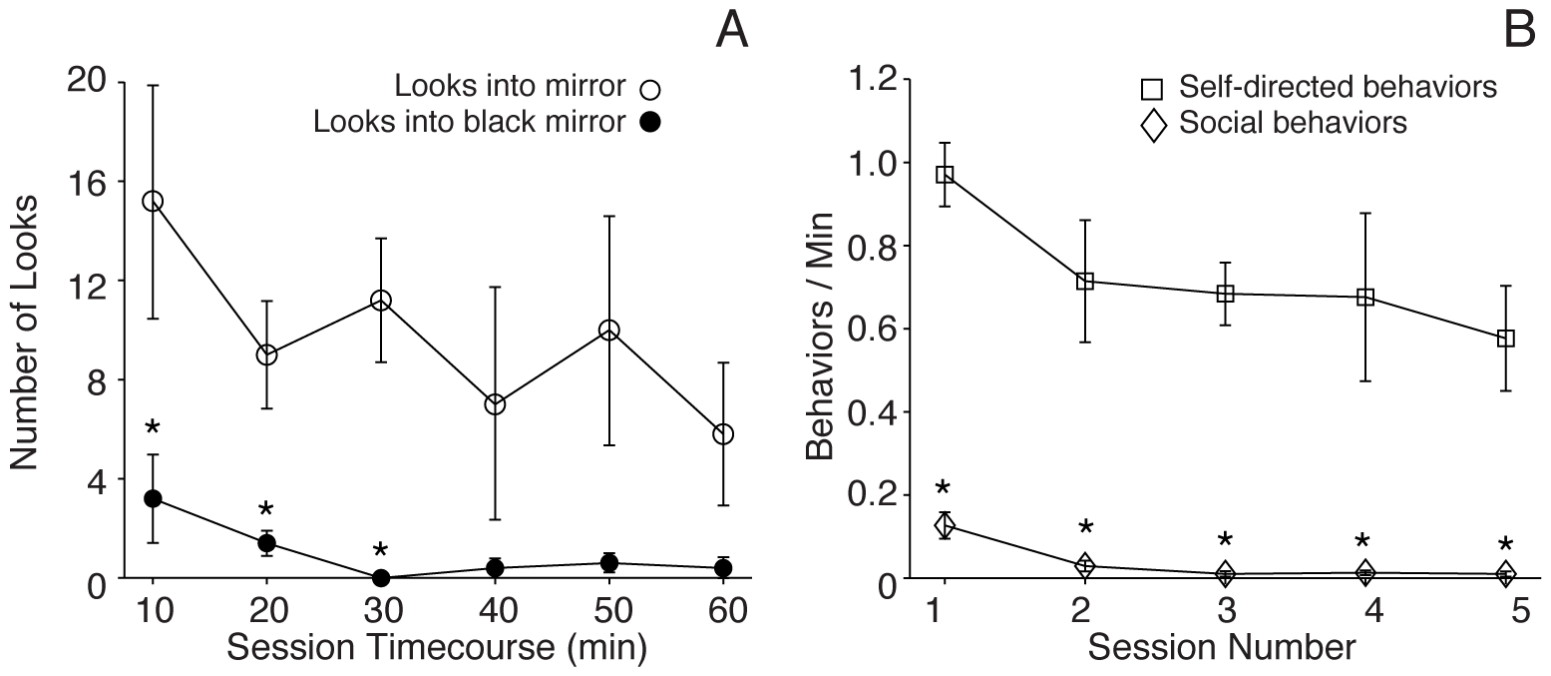
Quantification of mirror-directed behaviors during the first session and across five sessions. (A) Number of looks in the large mirror and large black-covered mirror during the first session. The one-hour session was broken up into 10-minute bins. (B) Number of looks into the large mirror and number of social behaviors directed at the large mirror per minute. The number of social behaviors in the last four sessions declined significantly (*p*<0.05) relative to the first. The standard bars represent standard errors and the asterisks indicate significance (*t*-test, *p*<0.05).

The number of looks into the mirror was significantly larger than the control during the first thirty minutes (*p*<0.05), declining slightly in the second half of the session (Fig. 3A). Throughout the first session the number of looks declined for the mirror and the control; the number of looks at the control was practically zero after 30 min. Few or no interactions were documented with the control in sessions 2–5. The monkeys appeared to simply ignore the black, non-reflective object.

One of the most important findings concerns the difference in the rate of occurrence of self-directed and social behaviors directed towards the large mirror (Fig. 3B). Social behaviors occurred at a lower rate (*p*<0.05) than self-directed behaviors. Fundamentally, unlike in previous reports in monkeys [1], their rate decayed significantly from the first to the second session (*p*<0.05), remaining at negligible levels in the subsequent three. This is similar to observations in chimpanzees [1], [20] and consistent with one of Anderson's [19] assertions that diminishing and ultimately extinguishing social behaviors are indicative of self-recognition during mirror tests. Lastly, the monkeys looked into the large mirror approximately once a minute, a rate that decreased slightly across all five sessions but the decline did not reach significance. The duration of the looks directed at the large mirror and its control were similar to the duration of the looks directed at the small mirror and control in Experiment 1 but, despite a trend for longer looks into the mirrors, the differences did not reach significance (Fig. 2B).

**Figure 4:**
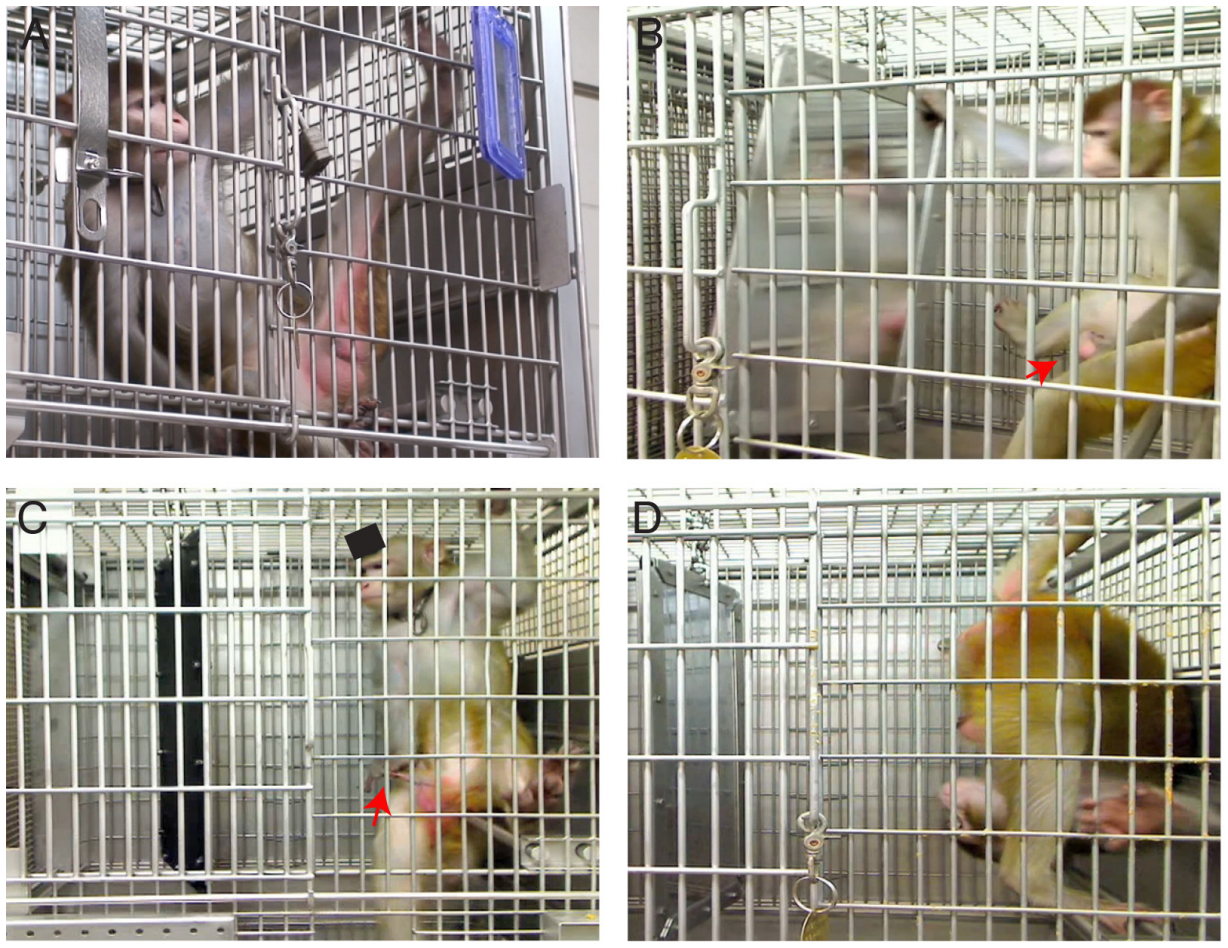
Example of monkeys examining their genital area in front of the mirrors. The red arrows point to the manipulation of the genitals (B,C). (D) Acrobatics such as this were commonly observed during inspection of genital area.

In addition, the monkeys used the mirrors extensively to look at their genitals (Fig. 4). This behavior was first observed after the head implant of one monkey was removed to avert a potential infection, after which he continued to use the small mirror but instead of observing and grooming the top of his head, he began inspecting and touching his genitals (see Movie S3); the implant was reattached later successfully. All five monkeys used the mirror to look at areas of their bodies they could not see directly. Sometimes they used one hand to hold the mirror in place (Fig. 4B) and moved or manipulated their genitals (Fig. 4B,C), while other times they performed acrobatics in what appear to be an effort to obtain a better view (Fig. 4D and Movies S4, S5). These observations are consistent with another of Anderson's [19] assertions concerning mirror-guided behaviors that are indicative of self-recognition and could be categorized under Povinelli et al.'s [20] classification of self-exploratory behavior used as a positive indicator of mirror self-recognition in chimpanzees. As shown in Figure 2C, the rate of looking at unseen areas, the genitals in particular, was ten times smaller in the absence of the mirror.

Notably monkeys that had not been implanted were not observed using the mirror suggesting that the implant constituted a relevant stimulus, a “super mark,” that prompted them to look. We confirmed this by observing the behavior of two monkeys the day after the implant was attached. After some hesitation both monkeys began to look at themselves in the mirror and to examine the area around the implant. Unequivocally more revealing was that they attempted to pull the head post off their heads while looking in the mirror, a behavior that subsided after a few attempts and was not observed again. Most importantly, these behaviors were mirror-guided and self-directed but never directed toward the reflection in the mirror.

## Discussion

Here we have shown that rhesus monkeys, though failing the mark test, demonstrate behaviors indicative of mirror self-recognition. They use the mirror to groom their head implants and inspect unseen areas of their bodies such as their genitals. Though we cannot objectively claim that these animals are self-aware, all the pieces are there to suggest that, in some form, they are.

If the ability to demonstrate self-recognition were innate, as suggested by Gallup [7], and could be explained solely on evolutionary grounds, one would expect that most, if not all members of a given species would or would not pass the test [21]. As it turns out, only a fraction of chimpanzees shows signs of mirror self-recognition [20], [21]. Furthermore, one would not expect a phylogenetic gap in the expression of this ability, a conclusion derived from the fact that gorillas fail to show signs of mirror self recognition and fail the mark test [20], while orangutans, though lower evolutionarily, do [22]; but see [23], [24] for positive evidence from two different gorillas. Note: even in children the proportion that exhibit self-recognition at a particular point in development varies as a function of intelligence level, cultural background, and type of self-recognition test administered [25]–[27].

A more likely explanation, however, is that behaviors indicative of mirror self-recognition are learned by establishing a contingency between self-produced movement and the reflection. The capability to learn and establish such a contingency and the form in which it is expressed is likely to vary across species. The question arises, therefore, as to the conditions that facilitate the establishment of the contingency.

Overall, the data are consistent with the saliency hypothesis [16], which postulates that an alteration in an individual's body must be highly salient to draw attention to the mirror image. Accordingly, the changes imposed on the appearance of monkeys in the standard mark test, as with more extensive markings in cotton-top tamarins [28], are not sufficient to draw the animal to touch the marks while looking in the mirror. The head implant, on the other hand, constitutes a relevant change that motivates the subject to use the mirror to inspect the area around it.

The sudden onset of self-directed behaviors in front of the mirror suggests that the monkeys either developed this ability *de novo* as a result of the surgery or were aware that they could see themselves in the mirror but were unable, perhaps due to gaze aversion, or uninterested in looking at themselves until a sufficiently relevant change took place - implantation of the head cap, therefore, simply triggered the display of this ability. Based on the length of the exposure to mirrors of these monkeys before they received the implants (all grew up with mirrors and were exposed to them constantly throughout their lives as part of their enrichment program), we conclude that the data are consistent with the latter.

We hypothesize that for the monkeys in this study the implant constituted a “super mark” that, coupled to their prior experience with the small mirror, the mobility of both mirrors, and the monkey's direct access to them, facilitated the manifestation of these behaviors. Future study should reveal what are the most effective experimental conditions, including mirror configurations and the time required to develop the contingency.

The mark test [1], therefore, is an inadequate measure of self-recognition for rhesus monkeys. A similar argument can be made for the results of studies of other species that rely heavily on audition or olfaction, as the mark test relies solely on vision, because they may reveal some form of self-recognition if tested differently [15], [29]. More fundamentally, the mark test may not be enough to reveal that members of a given species are self-aware [14].

These observations, taken together, demonstrate that rhesus monkeys do recognize themselves in the mirror and, therefore, have the fundamental elements to have the capacity to be self-aware. Accordingly, we conclude that behavioral differences between hominoids and lower primates are not the result of cognitive deficits in the latter, but rather of a different position on the underlying evolutionary continuity of mental functions [6], [15], [30].

## Materials and Methods

Five male rhesus monkeys (*Macaca mulatta*) 5–13 years of age that had been exposed to mirrors as part of their enrichment throughout most of their lives were studied. A sixth monkey, who had also received a head implant, showed no interactions with the mirror and thus was not included in the study. The mirror was two-sided, set in a plastic frame measuring 3×4.75 inches, hung outside the cage, and could be swiveled. All five subjects had been prepared for behavioral/electrophysiological experiments with a head implant, the area around which was cleaned before experiments with a dry cotton swab. The implant consisted of a block of acrylic (Ortho Resin, Justi Products, Oxnard, CA) ranging in size ∼(40 mm–100 mm×40–80 mm). The acrylic was blue in color and held (1) a lightweight titanium head post used for holding a water spout in front of the animal's lips during head-unrestrained oculomotor experiments and to restrain the head to clean and care for the area surrounding the implant [31], (2) connectors for the eye coils [32] used to record eye movements with the scleral search coil technique [33], and (3) a cylinder to insert microelectrodes into the brain for physiological recordings. Two implants, one with and one without a recording cylinder are shown in Figure S1. The implants were attached to the skull of the subjects with human grade titanium screws.

The animals were housed individually and provided double the space, 12.4 cu ft, typically provided for rhesus monkeys. Data were acquired in the room where the animals were housed. Video recordings followed the initial observations using a webcam without humans present. The animals continued to participate in their assigned experiments. Five one-hour sessions were videotaped for the two mirror sizes, small and large (12×24 inches set in a metal frame), and corresponding controls in which the mirrors were covered with black non-reflective plastic. The non-reflective controls were used to determine if similar behaviors took place when the monkeys could not see themselves.

The data were scored offline for self-directed and social behaviors in front of the mirror according to the categories outlined in Table 2. All behaviors scored were active and purposeful, that is the monkey either positioned the mirror with his hand to look at himself or moved to attain the appropriate angle for viewing himself. These are similar to the criteria used to characterize the behavior of chimpanzees [20]. Of particular importance were self-exploration, defined as manipulation of areas not visible without use of the mirror (e.g., the anal-genital area) used to classify animals as showing positive evidence of self-recognition, and social behaviors, aggressive or appeasing gestures suggesting that the monkey sees a conspecific [20]. Three observers, aware of the hypothesis being tested, viewed and scored the first group of data collected according to the behavioral categories listed in Table 2. The formula used by Povinelli et al. [20] was used to calculate reliability where the percentage of agreement between observers  =  total instances of agreement/total opportunities for agreement with L.C. Populin used as the standard for comparison. A congruency between the scoring of three observers exceeded 95% in the first group of video data obtained thus only one observer scored the remaining data. The small proportion of inconsistencies among the three observers primarily comprised the length of brief behaviors such as glances into the mirror; they were resolved by consensus after frame-by-frame review of the pertinent sections of the video record. All efforts were made to ameliorate suffering of the animals. Specifically, all procedures were approved by the University of Wisconsin Animal Care Committee and were in accordance with the National Institutes of Health *Guide for the Care and User of Laboratory Animals*.

## Supporting Information

Figure S1 Head implants. (A) Basic head implant used for behavioral experiments. The acrylic holds a titanium head post and two connectors for eye coils. (B) Head implant used for physiological experiments. A recording cylinder, 19 mm in diameter, has been added to the basic implant to allow the insertion of microelectrodes.
(1.35 MB TIF)

Movie S1 Self-directed behavior in front of the mirror. This movie shows a monkey waking up from a nap, then reaching for the small mirror outside his cage, positioning it to view himself, and grooming the area around the implant while looking at himself. A green mark used for the mark test, which he failed, is still visible on his left cheek. The view of the head implant has been blocked for discretion.
(0.33 MB MOV)

Movie S2 Typical use of the large mirror by monkeys. This movie shows a monkey using the large mirror inside his cage to view his neighbor and to examine himself. Note the position of his right leg, which is elevated thereby exposing his genital area. For nearly one minute the monkey observes himself without signs of social behaviors directed at the mirror.
(1.10 MB MOV)

Movie S3 This movie shows a monkey inspecting the lower part of his body and genitals using the small mirror. He looks over his shoulder to view his backside and genitals. Note that toward the end of the movie he reaches with his hand between his legs and pushes his genitals forward into view, confirming, therefore, that he is examining them in the mirror. This movie was recorded after the implant had been removed from this monkey.
(1.12 MB MOV)

Movie S4 Use of the big mirror to inspect genitals; two clips are shown in succession. First the monkey positions the mirror, orients and lifts his left, then grabs his genitals while looking attentively. Second, the monkey directly looks between his legs, then turns toward the mirror to view the same part of his body.
(0.89 MB MOV)

Movie S5 Monkey performing acrobatics in front of the mirror to view his backside and genitals. First the monkey looks between his legs while pushing his genitals with his hand. Second, he hangs upside down from the top of his cage while attempting to view his genital area from this angle.
(3.12 MB MOV)
