# Supplementary figures and images for: Rhesus Monkeys (Macaca mulatta) Do Recognize Themselves in the Mirror: Implications for the Evolution of Self-Recognition
Source: PLoS One. 2010 Sep 29;5(9):e12865. doi: 10.1371/journal.pone.0012865 (PMC2947497; doi:10.1371/journal.pone.0012865)

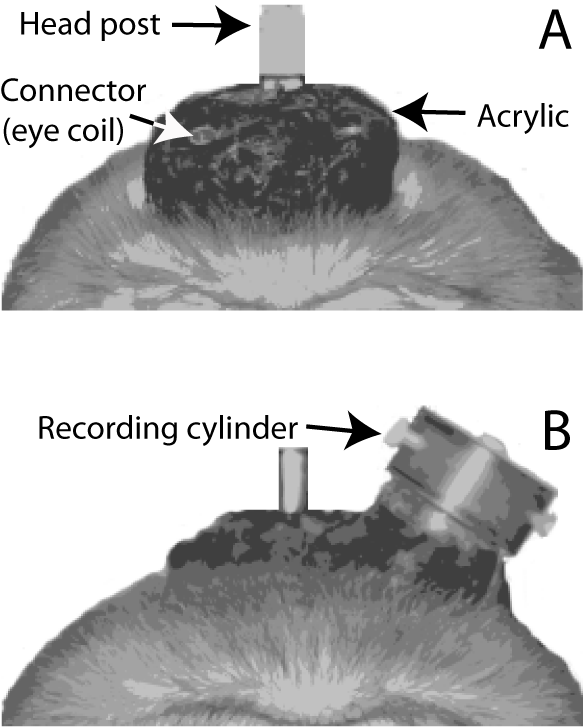

Supplement: Figure S1 — Head implants. (A) Basic head implant used for behavioral experiments. The acrylic holds a titanium head post and two connectors for eye coils. (B) Head implant used for physiological experiments. A recording cylinder, 19 mm in diameter, has been added to the basic implant to allow the insertion of microelectrodes. (1.35 MB TIF) [file pone.0012865.s001.tif]
